# Molecular tests support the viability of rare earth elements as proxies for fossil biomolecule preservation

**DOI:** 10.1038/s41598-020-72648-6

**Published:** 2020-09-23

**Authors:** Paul V. Ullmann, Kristyn K. Voegele, David E. Grandstaff, Richard D. Ash, Wenxia Zheng, Elena R. Schroeter, Mary H. Schweitzer, Kenneth J. Lacovara

**Affiliations:** 1 Department of Geology, Rowan University, Glassboro, NJ USA; 2 Department of Earth and Environmental Science, Temple University, Philadelphia, PA USA; 3 Department of Geology, University of Maryland, College Park, MD USA; 4 Department of Biological Sciences, North Carolina State University, Raleigh, NC USA; 5 Department of Geology, Lund University, Lund, Sweden; 6 North Carolina Museum of Natural Sciences, Raleigh, NC USA; 7 Museum of the Rockies, Montana State University, Bozeman, MT USA; 8 Jean and Ric Edelman Fossil Park At Rowan University, Rowan University, Mantua Township, NJ USA

**Keywords:** Palaeontology, Geochemistry

## Abstract

The rare earth element (REE) composition of a fossil bone reflects its chemical alteration during diagenesis. Consequently, fossils presenting low REE concentrations and/or REE profiles indicative of simple diffusion, signifying minimal alteration, have been proposed as ideal candidates for paleomolecular investigation. We directly tested this prediction by conducting multiple biomolecular assays on a well-preserved fibula of the dinosaur *Edmontosaurus* from the Cretaceous Hell Creek Formation previously found to exhibit low REE concentrations and steeply-declining REE profiles. Gel electrophoresis identified the presence of organic material in this specimen, and subsequent immunofluorescence and enzyme-linked immunosorbant assays identified preservation of epitopes of the structural protein collagen I. Our results thereby support the utility of REE profiles as proxies for soft tissue and biomolecular preservation in fossil bones. Based on considerations of trace element taphonomy, we also draw predictions as to the biomolecular recovery potential of additional REE profile types exhibited by fossil bones.

## Introduction

Ancient biomolecules are sought after by the paleontologic community as a uniquely-informative resource into the phylogenetic relationships and paleobiology of extinct organisms. As two recent examples demonstrate, biomolecular data derived from vertebrate fossils have been used to independently test the phylogenetic position of non-avian dinosaurs within Archosauria (e.g.,^1,2^) and track structural adaptations in keratin over time during the early evolution of paravians^3^. Biomolecules present in bones experience varying degrees of alteration and degradation through fossilization and diagenesis (e.g.,^2,4,5^), during which time the mineral component of bone also undergoes elemental, isotopic, and mineralogical alterations^6–8^.

Despite decades of independent research on the preservation of endogenous elemental and isotopic signatures (e.g.,^6,9–12^) and still-soft tissues containing remnants of original biomolecules (e.g.,^1–5,13–22^), no study has directly tested for a link between these research foci by performing comparative analyses on the same fossil specimen. As a result, the relationship between retention of original isotopic signatures and/or trace element signatures to retention of endogenous soft tissues and/or molecules in biomineralized tissues remains unknown.

Rare earth elements (REE) are ubiquitous in fossil bones^7^ and have the potential to inform on diagenetic alteration. The concentrations of REE in a fossil bone reflect the extent of diagenetic pore fluid interactions the specimen has experienced^23–28^. Because groundwater moving through bone is the primary delivery mechanism of REE, the concentrations of REE in a specimen are thought to correlate with the duration and extent of pore fluid interactions with a bone over its burial history^7,24,25,27,29^. The spatial distribution of REE within a fossil bone also provides a detailed record of diagenetic alteration^24,26,28,30^; therefore, REE concentration profiles may elucidate geochemical conditions favoring biomolecular preservation, or lack thereof, especially if correlated with other indicators of exceptional preservation.

Retention of endogenous isotopic signatures and early diagenetic REE profiles is predicted to be positively correlated to biomolecular preservation because each presumably requires minimal chemical alteration of bone through diagenesis (cf., Trueman and Martill^31^, Schweitzer^32^, and references therein). Degradation of bone crystallites due to inorganic or organic (i.e., enzyme-driven) dissolution exposes the proteinaceous matrix, leaving it vulnerable to hydrolysis, oxidation, and other destructive forces^31,33^. Conversely, decay of the fibrous protein matrix exposes otherwise passivated (“shielded”) bone crystallite surfaces to geochemical gradients in permeating pore fluids^31,33^. This molecular-level codependency forms the basis of the historical “mutual protection” theory of bone fossilization (e.g.,^31,33–35^).

**Figure 1:**
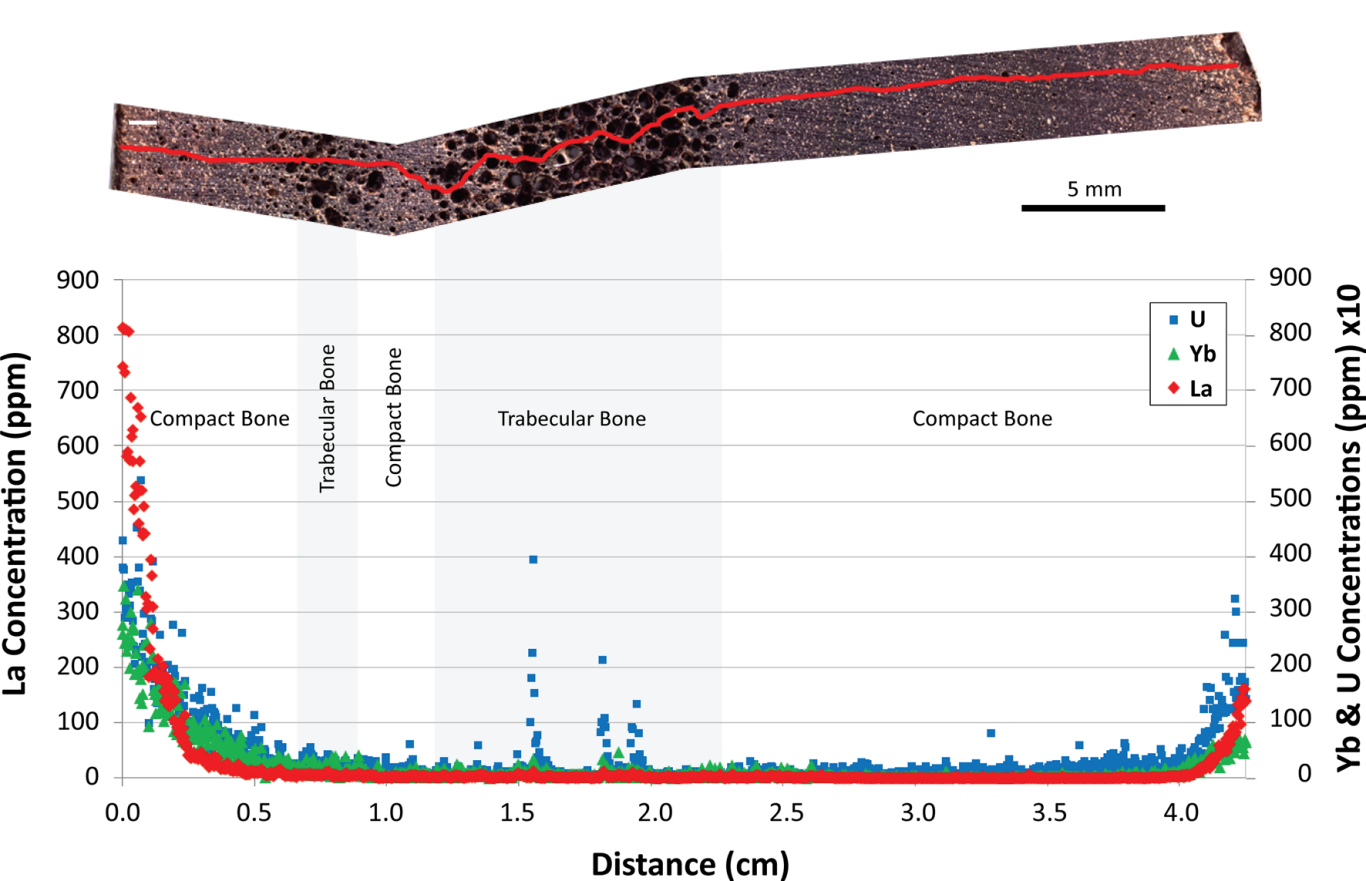
Intra-bone concentration gradients of lanthanum (La, red diamonds), ytterbium (Yb, green triangles), and uranium (U, blue squares) for *Edmontosaurus annectens* fibula SRHS-DU-231, tested for biomolecular preservation in this study. Profiles cross the entire diameter of the bone. Laser track denoted by the red line in the bone cross section. High-porosity trabecular tissue regions are shaded in gray. Scale as indicated in upper right. Reproduced, with permission from *Geochimica et Cosmochimica Acta*, from Ullmann et al.^43^.

A rarely considered corollary of “mutual protection” is that if one encounters excellent preservation of the crystal portion of a bone (i.e., retention of histological microanatomy, relatively unaltered early-diagenetic trace element signatures), it is reasonable to propose that the bone may also yield traces of its original biomolecular composition. Trueman et al.^24^ first proposed this trace-element extension of “mutual protection” theory, suggesting that REE concentration-depth profiles may be a proxy for biomolecular preservation in fossil bones. Specifically, they predicted that bones exhibiting low overall REE concentrations and steeply declining REE concentrations with cortical depth, such as the specimen studied herein (Fig. 1), should be the best candidates to retain endogenous biomolecules. They posit this because these patterns signify the least interaction with pore waters (the primary source of hydrolytic damage to biomolecules). If data were found in support of these hypotheses, it would provide molecular paleontologists with a tool for screening suites of fossils prior to more costly and time-intensive biochemical analyses (e.g., protein extractions, mass spectrometry).

To test these correlations, we compared intra-bone REE patterns with the results of immunoassays against the dominant bone protein collagen I from the same bone, SRHS-DU-231, a fibula of the hadrosaurine dinosaur *Edmontosaurus annectens* from the Standing Rock Hadrosaur Site (SRHS) in Corson County, South Dakota. We complement analyses of this specimen with discussion of correlations between REE patterns and soft tissue recovery in eight additional SRHS bones previously subjected to demineralization assays^36^.

### Geologic context

*Edmontosaurus annectens* bones at SRHS are preserved in a mass-mortality assemblage near the base of the Maastrichtian Hell Creek Formation^37^. Bones here are entombed in a 30 cm thick, silty, sandy, mottled, organic-rich mudstone interpreted to represent distal deposition of crevasse splay/flood sediments into a shallow, reducing, coastal-plain pond^37,38^. Colson et al.^38^ and Ullmann et al.^37^ provide further details on the sedimentology, stratigraphy, and taphonomic context of the fossil bones at SRHS.

## Results

### Polyacrylamide gel electrophoresis (PAGE)

**Figure 2:**
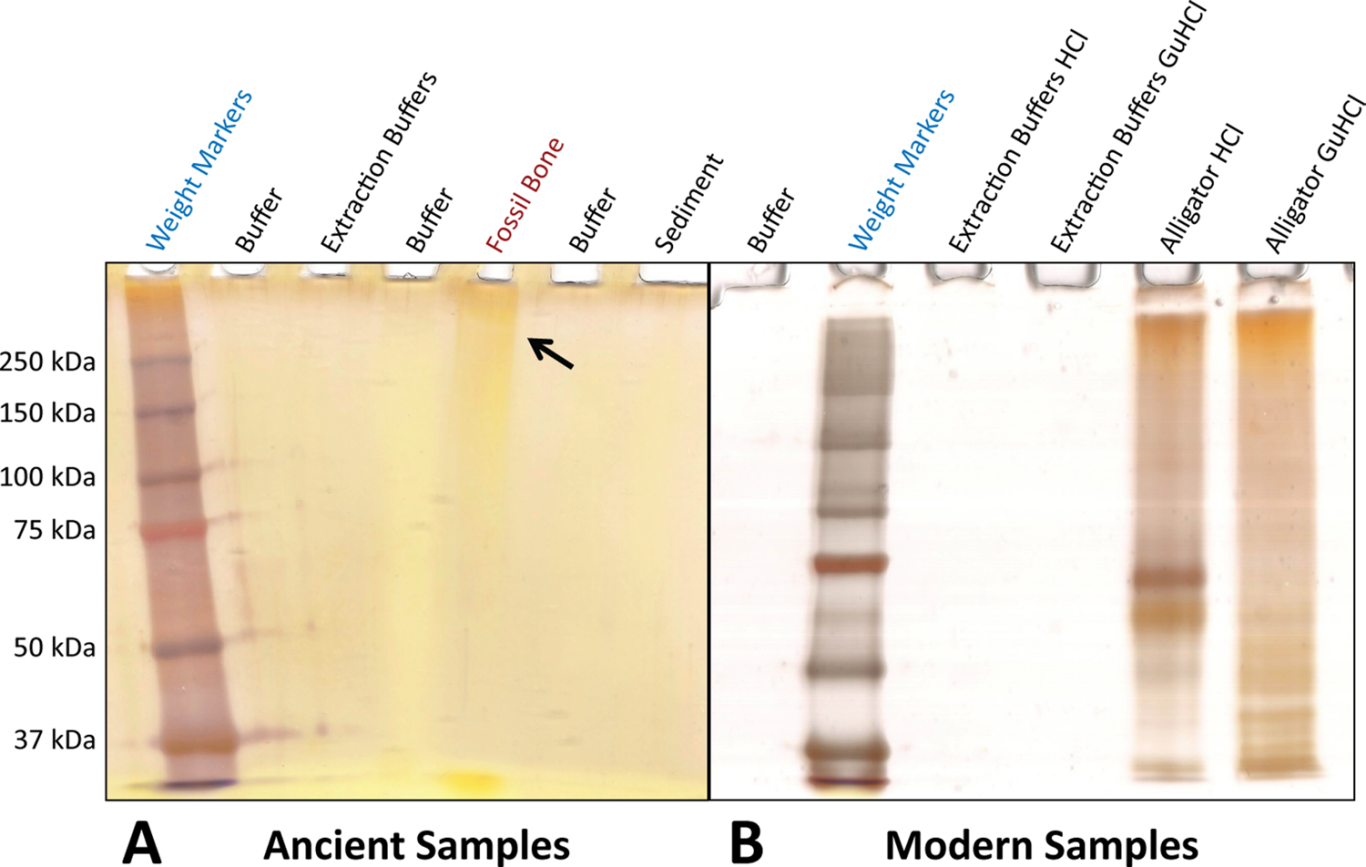
Polyacrylamide gel electrophoresis (PAGE) with silver staining of fossil and modern GuHCl extracts. (**A**) Fossil bone (*Edmontosaurus* fibula SRHS-DU-231) and sediment GuHCl extracts were loaded at 4 mg of extraction yield per lane and extract resuspension buffers were run as a negative control. Orange staining is seen as a smear at high molecular weights in the fossil bone GuHCl lane (arrow). Dark brown bands in the buffer lane adjacent to the molecular weight markers were caused by slight carryover of the weight markers solution during loading. (**B**) Silver-stain results for modern control *Alligator* HCl and GuHCl extracts loaded at 20 µg/lane.

Protein extractions resulted in two chemical extract samples for each specimen and control, an ammonium bicarbonate (ABC) extract and a guanidine hydrochloride (GuHCl) extract (see Methods and the Supporting Information). When silver-stained, separated GuHCl extracts of *Edmontosaurus* fibula SRHS-DU-231 exhibited high molecular weight components in fossil bone lanes (Fig. 2A). Staining was visible as an orange smear that darkened upward from a molecular weight of ~ 150 kDa (Fig. 2A). No staining occurred in co-extracted sediment or buffer control lanes. Similarly-treated extant *Alligator* bone (extracted in a different, isolated lab with separate, dedicated reagents and equipment) exhibited both distinct bands and broad smears through the entire molecular weight range examined (Fig. 2B).

Fossil bone and sediment ABC extracts contained dark brown substances, which we interpret to likely be humic substances, as these are commonly co-extracted with proteins from fossil bones^39^. After electrophoresis but before silver-staining, gels containing ABC extracts exhibited dark (pre-development) coloration derived from these humics, usually visible as a light brown-orange hue (S1 Fig.). This coloration was consistently darker in resuspended bone pellet lanes than in initial bone extract lanes (see Supporting Information). Sediment ABC lanes also exhibited similar humics-derived pre-development coloration, seen as a faint, diffuse smear at middle molecular weights with a distinct band at ~ 65–70 kDa (S1 Fig.). Such pre-development coloration was restricted to ancient (fossil and sediment) samples; it was not observed in modern control assays.

Whereas humics-derived pre-development coloration in fossil bone ABC lanes was faint across all molecular weights (S1 Fig.), silver-staining intensified the color as a dark smear of breakdown products across all molecular weights. This trend was more intense at low molecular weights (seen as greater darkening < 20 kDa; S1 Fig.). These staining regions contrast those of modern *Alligator* ABC extracts, for which development revealed a dark band at ~ 60–65 kDa and a dark smear primarily across high molecular weights (> 37 kDa; S1 Fig.). Gel lanes loaded with sediment ABC extracts exhibited high molecular weight staining after development, seen as a yellow-orange color at molecular weights > 50 kDa. These lanes also exhibited darkening of the single pre-development band at 65–70 kDa (S1 Fig.); humic substances within the sediment extract thus bound silver. After development, the extraction blank also exhibited a very faint but distinct band at the same molecular weight as that in the sediment, ~ 65–70 kDa (S1 Fig.).

### Enzyme-linked immunosorbant assay (ELISA)

**Figure 3:**
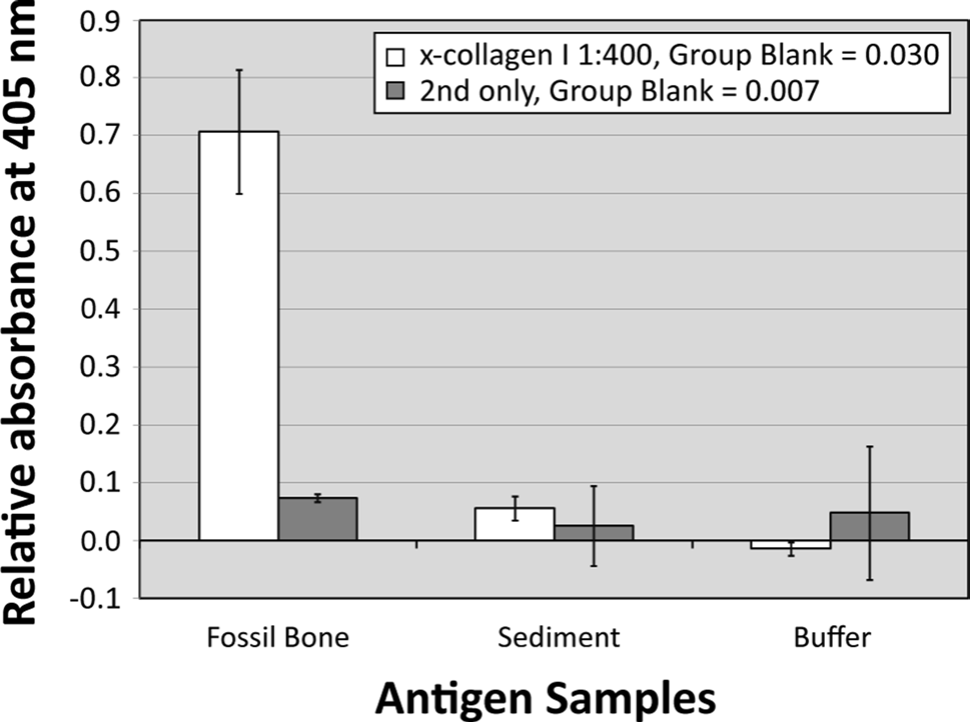
Enzyme-linked immunosorbant assay results. Fossil bone (*Edmontosaurus* fibula SRHS-DU-231) and sediment ABC extracts were plated at 200 mg of pre-extracted mass/well, and extract resuspension buffers were plated as a negative control. White columns represent absorbance values at 240 min with incubation in rabbit anti-chicken collagen I antibodies at a concentration of 1:400. Dark gray columns represent absorbance values for accompanying secondary-only controls. Error bars represent one standard deviation from the mean absorbance value for each sample.

ABC extracts of *Edmontosaurus* fibula SRHS-DU-231 incubated with anti-chicken collagen I antibodies (see Supporting Information) yielded absorbance values over 500% those of associated sediment and all other negative controls in multiple replicates (Fig. 3, S2 Fig.). These results support the presence of collagen I in the dinosaur extracts by standard ELISA signal acceptance criteria (positive ≥ two times background levels; e.g.,^40,41^). No similar signal was observed in co-extracted sediment or buffer control wells. Fossil extracts in multiple repeated ELISAs consistently exhibited reduced absorbance values in comparison to modern controls, in which *Alligator* ABC samples normally reached saturation (~ 3.0) by ~ 120 min. After the same amount of time, SRHS-DU-231 extracts reached an absorbance of 0.37 (S3 Fig.). Though this represents nearly an order of magnitude difference, absorbance by the fossil extract was still over an order of magnitude greater than those of the sediment (0.03) and negative controls (-0.01), thus surpassing the positive detection threshold.

### Immunofluorescence

**Figure 4:**
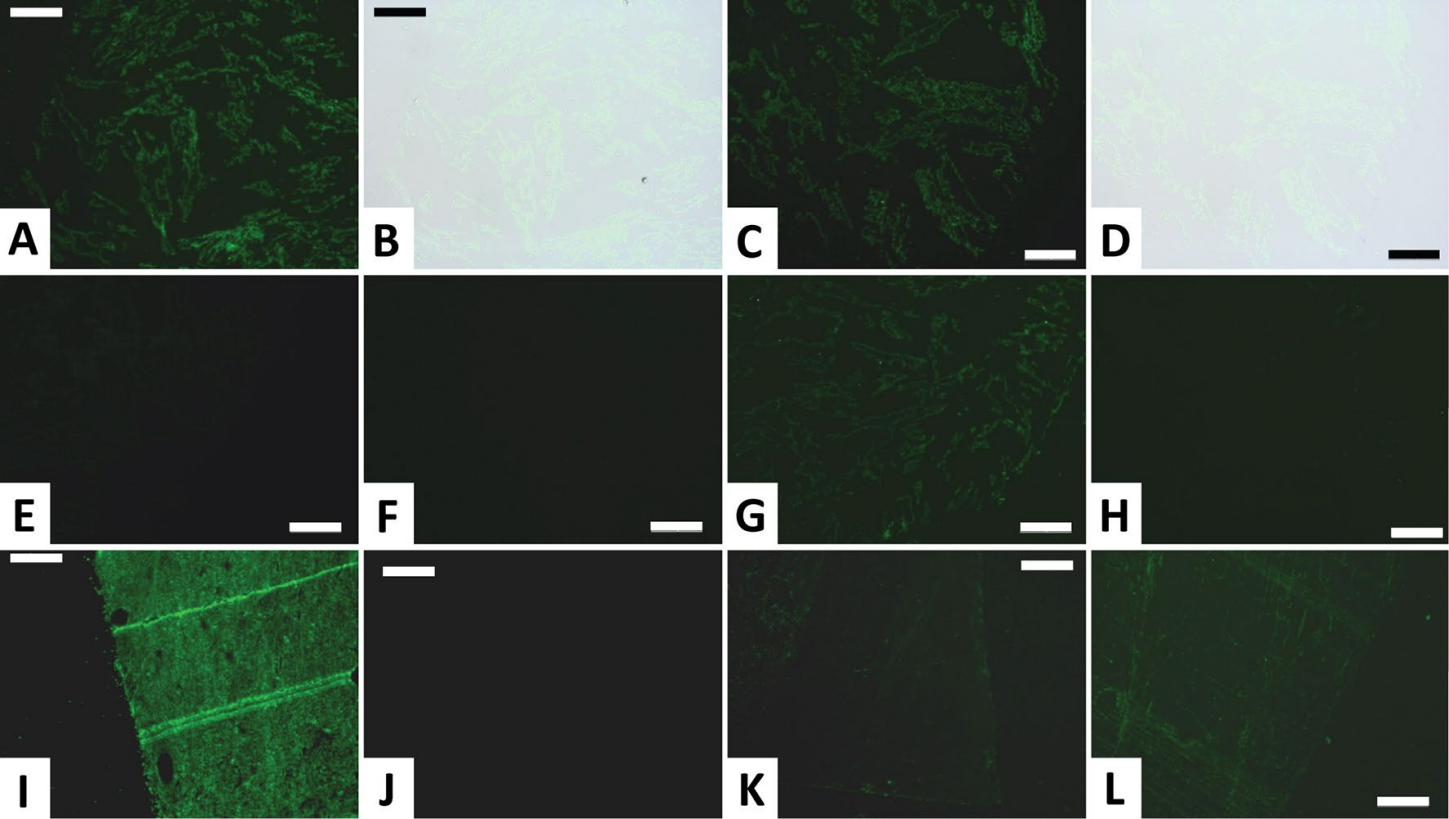
In situ immunofluorescence results. Localization of collagen I in demineralized cortical fragments of *Edmontosaurus annectens* (fibula SRHS-DU-231; **A–H**) and modern *Alligator* (**I–L**). (**A–D**) *Edmontosaurus* demineralization products incubated with antibodies against chicken collagen I; (**A**) and (**C**) show FITC-labeled antibody-antigen complexes in two tissue sections observed under fluorescent light, and (**B**) and (**D**) are overlay images showing that binding locations correspond to the location of tissue fragments within the embedding resin. All remaining images (**E–L**) show FITC-labeled antibody-antigen complexes under fluorescent light. (**E**) Secondary-only control section that was not exposed to primary antibodies, to control for non-specific binding of secondary antibodies. (**F**) Section exposed to anti-chicken collagen I antibodies that were first inhibited with chicken collagen (prior to incubation). (**G**) Section digested with collagenase for 1 h prior to exposure to anti-collagen I antibodies. (**H**) Section digested with collagenase for 6 h prior to exposure to anti-collagen I antibodies. (**I**) Modern *Alligator* tissue section incubated with anti-chicken collagen I antibodies. (**J**) Secondary-only *Alligator* control section that was not exposed to primary antibodies. (**K**) *Alligator* section exposed to anti-chicken collagen I antibodies that were first inhibited with chicken collagen. (**L**) *Alligator* section digested with collagenase for 1 h prior to exposure to anti-collagen antibodies. All section images were taken at 40X and 200 ms integration. All scale bars equal 50 µm.

Demineralized fibula SRHS-DU-231 cortical bone reacted positively to polyclonal anti-chicken collagen I antibodies in in situ immunofluorescence assays (Fig. 4A–D). Though clearly diminished relative to modern *Alligator* positive controls (compare Fig. 4A,I), fluorescence was clearly localized to tissue sections and no fluorescence was found in the slide background. To control for spurious binding of secondary antibodies or the FITC label, we also conducted assays omitting the primary antibody while keeping all other parameters identical. Inhibition (blocking epitopes with excess collagen) and collagenase digestion assays were also performed as specificity controls; these assays, respectively, control for non-specific paratopes in the polyclonal primary antibodies and non-specific binding of the primary antibodies to molecules other than the target protein. Secondary-only and inhibition specificity controls exhibited extremely reduced or no fluorescence (Fig. 4E,F,J,K). Enzymatic digestion controls involved digesting tissues with 1 mg/ml collagenase treatment prior to antibody exposure. This control also provides a secondary validation of the presence of protein fragments, as collagenase will not digest other proteins^35^. Digestion with this enzyme initially resulted in increased signal intensity (1 h digestion, Fig. 4G, L). When digestion times were increased from 1 to 6 h, dramatically reduced binding was observed (Fig. 4H). This result confirms the fossil antigens are structurally consistent with collagen (i.e., have abundant X-Gly bonds uncommon in other proteins^42^).

## Discussion

Our previous analyses of bones from SRHS found them to be well-preserved at the macro-scale^37^ and to commonly yield osteocytes and soft tissue microstructures (vessels and fibrous/proteinaceous matrix) upon demineralization^36^. Further, our REE data (e.g., Fig. 1) indicated a simple diagenetic history for SRHS bones that involved a brief period of primary trace element uptake relative to many other similar-age fossils^43^. Despite a lack of permineralization, pore fluid replenishment appears to have been impeded following early diagenesis, perhaps due to low permeability of the fine grain host matrix^43^. Minimal trace element uptake by these bones signifies minimal chemical alteration from their original state; therefore, *Edmontosaurus* bones from SRHS were predicted to be favorable candidates for preservation of endogenous biomolecules^43^, in accordance with Trueman et al.^24^.

We began by conducting polyacrylamide gel electrophoresis (PAGE) assays, which separate components of extracts by molecular weight. Though this technique is less sensitive than ELISA, it enables recognition of denatured epitopes^44^. Staining of electrophoretically-separated extracts with silver identified the presence of organics in *Edmontosaurus* fibula SRHS-DU-231 that differed in molecular weights from organics (likely humics) in the surrounding sediment (Fig. 2 and S1 Fig.). These results indicate that the bone organics do not represent contamination from the sediment or laboratory reagents. The smearing pattern found for both fossil GuHCl and ABC extracts is similar to that encountered in previous PAGE assays of fossil bone^1,15,45^. Specifically, the high molecular weight smear seen in the fossil bone GuHCl extract (Fig. 2) is potentially suggestive of diagenetically cross-linked molecules^15^ and smearing at lower molecular weights in fossil bone pellet ABC lanes (S1 Fig.) is suggestive of molecular fragmentation/degradation (cf.,^34,46^). However, the precise identity of the organic materials cannot be determined by this assay alone, especially without clear banding that could be assigned a particular molecular weight.

Pre-development coloration of fossil and sediment lanes in gels loaded with ABC extracts is best attributable to the presence of humic substances in sediment and fossil bone extracts^39^. Though the color is also suggestive of iron and the sediment and fossils derive from a setting rich in iron^37^, treatment of extracts with an iron chelator (10 mM pyridoxal isonicotinic hydrazide in 50 mM NaOH) did not prevent pre-development coloration (Supporting Information). Moreover, fixation in 50% methanol slightly reduced but did not eliminate the pre-development coloration. Thus, humics appear to be a more plausible explanation than iron for pre-development coloring of lanes within gels loaded with ABC extracts. Lack of pre-development coloration in gels loaded with GuHCl extracts implies that GuHCl did not co-solubilize humic substances as effectively in these specimens as ABC.

The band seen at ~ 65–70 kDa in extraction blank lanes (S1 Fig.) may represent a minor contaminant in the extraction buffers or slight carryover of an organic substance present in the sediment extracts. We suggest it is more likely a product of carryover from the sediment lane as this band was never seen in fossil bone lanes which would otherwise also show the band if it represents an extraction buffer contaminant. Although the molecular weight (MW) of the substance is suggestive of common laboratory contaminants such as albumin (MW = 66.5 kDa^47^) or alpha keratin (MW = 66 kDa^48^), its identity cannot be determined with certainty by silver staining alone. Ultimately, whatever the substance is, it is not present in the fossil extracts and therefore has no pertinence to the results of our immunoassays.

To test whether organics in SRHS-DU-231 identified by silver staining included the most abundant bone protein, collagen I, we performed two immunoassays: ELISA and in situ immunofluorescence. Immunoassays provide remarkable sensitivity and specificity in comparison to less-specific, outdated techniques (e.g., amino acid analysis, racemization; see reviews by Schweitzer et al.^44^ and Cleland and Schroeter^49^). Whereas ELISA tests for antibody binding to solubilized epitopes in native confirmation in chemical extracts (and therefore provides very high sensitivity^44^), immunofluorescence provides the advantage of localizing epitopes of specific proteins in native confirmation to tissues^15,44^. The two assays thus provide independent, complementary information which, when performed with appropriate controls, can effectively evaluate the potential presence and endogeneity of biomolecules in fossil tissues^44^. To account for non-specific binding, we: (1) employed complementary controls, including sediment, secondary-only, inhibition, and digestion tests, and; (2) used modern, chromatography-purified, antigen-specific antibodies (from Millipore and U.S. Biological). Collectively, these measures can effectively eliminate non-specific binding, identify any potential false positives, and provide unequivocal support for true positive reactions (e.g.,^3–5,17,21,22^).

Immunoassays positively identified collagen I within demineralization products of *Edmontosaurus* fibula SRHS-DU-231 (Fig. 4A–D) and in chemical extracts from cortical samples of this specimen (Fig. 3). In both in situ immunofluorescence and ELISA, reactivity was only observed when fossil tissues or extracts were incubated with primary antibodies; sediment and secondary-only controls were negative (Fig. 3 and Fig. 4E, J). Because all parameters were kept identical, only the presence or absence of specific primary antibodies determined positive results. Thus, binding cannot be ascribed to humics, contamination, or spurious binding of secondary antibodies. Moreover, both inhibition and (3 h and 6 h) digestion controls demonstrate the specificity of antibody binding to collagen I (Fig. 4F–H, K, L). Initial increase in fluorescence in short (1 h) digestion assays has been previously reported and attributed to initial exposure of physically-shielded epitopes within molecular condensation products or aggregates^45,48^ or to a need for longer digestion because of diagenetically-induced crosslinks (e.g.,^50^). Our results are thus entirely consistent with detection of endogenous collagen I in SRHS-DU-231. Thus, we have documented the first evidence in support of Trueman et al.’s^24^ proposal of a link between steep REE depth profiles^43^ and biomolecular recovery potential.

Demineralization of cortical bone from SRHS-DU-231 yielded diverse cellular and tissue microstructures^36^. Based on the morphology of fluorescing tissues in our immunofluorescence assays (e.g., Fig. 4A), antibodies are most likely binding to semi-translucent/white fragments of tissue morphologically consistent with fibrous matrix visible by optical microscopy (Fig. 3 of ref.^36^). Though immunoassays have only been performed on one bone, that many other SRHS bones yielded identical, abundant, fragments of fibrous matrix upon demineralization^36^ suggests collagen I is likely to also be preserved in other bones from this locality.

Burial and diagenetic processes influencing the REE composition of SRHS bones likely also contributed to preservation of soft tissues and collagen in these specimens. First, rapid burial by flooding^37^ yielded consistent trace element composition among bones^43^ and minimized degradation during aerial exposure (e.g., by UV radiation-induced weathering^8,51^). Rapid burial is widely considered essential for soft tissue preservation in the fossil record^32,52,53^. Second, burial in a chemically-stable, circum-neutral pH, low-energy environment^37^ likely restricted the development of varied, complex REE compositions^43^ and minimized degradative processes acting on the bones and their soft tissues (i.e., abrasion, exposure to varied chemical regimes^54,55^). Third, chelation of metal ions in the reducing burial setting (as indicated by abundant plant matter and early-diagenetic siderite concretions within the bonebed^36,37^) may have minimized oxidative damage to collagen and other biomolecules^53^. Fourth, the argillaceous host matrix that limited pore fluid replenishment and therefore REE uptake^43^ may also have mitigated hydrolysis of biomolecules and physically restricted influx of microbial decomposers (e.g.,^56^). Abundant clay in the host matrix^37^ may also have mitigated degradation of soft tissues by adsorbing and thereby inactivating microbial enzymes^57,58^. Finally, waterlogged conditions combined with low permeability of the host matrix^37^ may have produced local zones of anoxia that could have further limited microbial activity and oxidation of biomolecules (e.g.,^34,59^).

Trueman et al.^24^ hypothesized that the REE composition of fossil bones should be linked with biomolecular recovery potential because the same pore fluid interactions that cause recrystallization and lead to REE uptake will also affect the biomolecular components of bone through hydrolysis, solubilization, and mobilization of autolytic enzymes and microbial decomposers. Though the high adsorption capacity of bone apatite crystallites^25^ may promote primary REE uptake during early diagenesis as Trueman et al.^24^ suggested, additional, protracted uptake over geologic timescales of burial can potentially alter and/or obscure original concentration-depth profiles. Such altered profiles can: (1) provide biased “fossilization rate” estimates from diffusivities^60^, and; (2) yield erroneous Lu–Hf isotopic ages^27,61,62^. Thus, even bones with as low REE concentrations and simple depth profiles as SRHS specimens are unlikely to facilitate accurate characterization of the duration of early-diagenetic recrystallization (“fossilization”) in an absolute sense.

**Figure 5:**
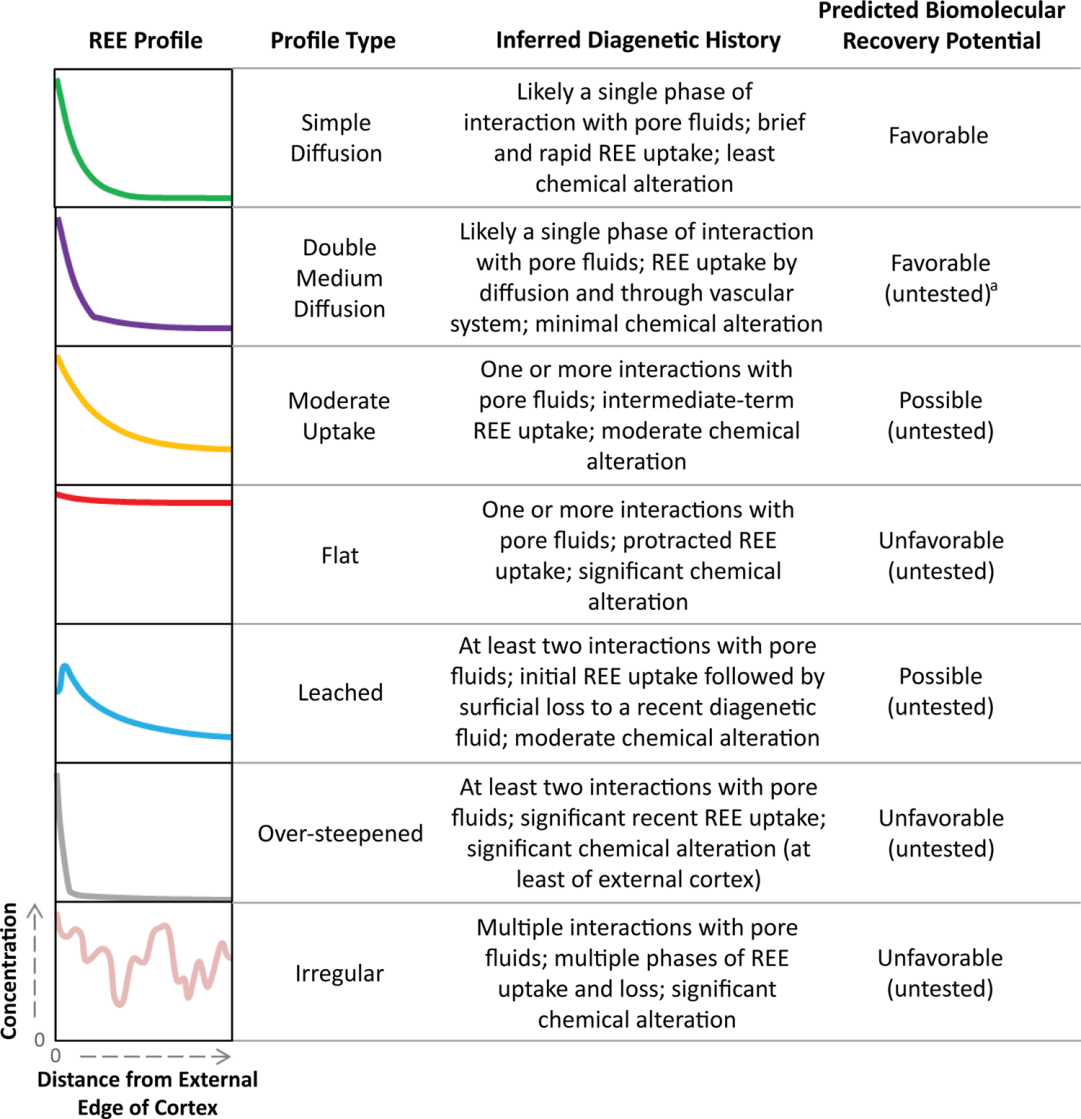
Predicted biomolecular recovery potential for bones with varied diagenetic histories inferred from REE profiles. Left-most column presents generalized REE profiles with depth into the bone from the external cortex margin increasing to the right and elemental concentration increasing upward. A double medium diffusion profile is portrayed as modeled by Kohn^70^. ^a^Though bones with double medium diffusion REE profiles have not yet been tested for biomolecular preservation, two SRHS bones exhibiting this profile type have successfully yielded microstructures upon demineralization that are morphologically consistent with modern analogs (osteocytes, blood vessel fragments, fibrous matrix)^36^. All SRHS bones exhibit simple diffusion or double medium diffusion profiles alike those depicted in the top two rows^43^.

What can be discerned from trace element taphonomy with confidence, however, is the overall relative extent of diagenetic alteration of a bone. Crucially, this should also be what governs the degradation of bone soft tissues and their constituent biomolecules. In short, “simple diffusion” profiles (sensu Trueman et al.^24^) may yield erroneous isotopic ages but still denote simple long-term diagenetic histories that are favorable for biomolecular preservation (Fig. 5).

Although debate continues over the precise mechanics of REE uptake in bone (e.g.,^25,27,28,60–63^), uptake mechanisms are irrelevant to the use of REE composition as a viable proxy for biomolecular recovery potential. Though identifying whether “simple diffusion” profiles (such as those found in most SRHS bones^43^) result from rapid REE uptake in early diagenesis followed by closed system behavior or from continual minor uptake of additional REE over geological timescales (e.g.,^27^) is vitally important when attempting to constrain the geochemistry of the original depositional environment, a fossil specimen’s potential for biomolecular preservation depends on its entire diagenetic history, not just the initial geochemical regime to which it was exposed. Therefore, we suggest that the overall level of trace element concentrations and the general character of REE profiles are the most important factors for predicting the likelihood of molecular recovery in fossil bones. Evident fractionation among REE (i.e., lower LREE/HREE ratios in the internal cortex relative to the external cortex, as in SRHS specimens^43^) may also be worth consideration as an important predictor as it signifies brief and/or spatially-restricted diffusion. Even if REE are continually incorporated through late diagenesis, models suggests that this usually occurs at very minor levels (likely < 0.2 ppm/Ma^27^). It thus constitutes a slow, relatively inconsequential process rather than a rapid, strong geochemical shift that would be more apt to degrade bone organics. Moreover, even if leaching, late-diagenetic uptake, or other processes alter initial REE depth profiles to the degree that they no longer accurately record chemical signals from the original environment of burial (e.g.,^27,64^), the spatial distribution of REE within the specimen will still characterize the complexity of interactions the fossil had with pore fluids over the duration of burial because these interactions leave identifiable chemical signatures (e.g.,^12,64–66^). Thus, no matter the duration or mechanism(s) of uptake, REE profiles constitute a valuable record of the history of diagenetic regime(s) controlling initial bone alteration and long-term stability.

In agreement with reports by prior authors, our previous study on the trace element composition of these specimens^43^ found that porosity clearly governs effective diffusion of trace element ions through bone; porosity and permeability facilitate trace element uptake and hence alteration. In principle, this suggests that denser, less-porous fossil bone tissue (i.e., in the middle cortex of limb bones) may generally be more resilient to alteration (cf.,^6^) and thus a more favorable target for biomolecular assays. However, we reiterate that cancellous bone tissues may also be worthy of molecular investigation as they have also occasionally yielded cells and soft tissues (e.g.,^16,36^).

Based upon these and prior data, we predict that specimens that incorporated REE primarily in early diagenesis and then became effectively closed systems (i.e., through early cementation of sediments and/or burial in low-permeability sediments, early permineralization, or encasement in early-diagenetic concretion) would be the best candidates to retain biomolecules because of long-term equilibration with the diagenetic environment (Fig. 5). However, a mounting body of evidence suggests that such specimens may be uncommon^27,61,62^. *Edmontosaurus* bones from SRHS and two Cretaceous bones analyzed by Koenig et al.^30^ appear to be the product of such simple diagenetic histories^43,61^, but it is common for fossil bones to exhibit considerably more complex diagenetic histories. Specimens which display evidence of trace element leaching (e.g., Fig. 2b of ref.^27^), secondary uptake phases (e.g., Fig. 2c of ref.^27^), or yet more complex diagenetic histories involving multiple exposures to groundwaters of varying compositions (e.g., Fig. 2e,f of ref.^27^; Fig. 9g of ref.^28^) would be expected to have lower likelihood of biomolecular recovery because any of these processes would involve re-exposure to new pore fluids later in diagenesis that could actively degrade soft tissues (Fig. 5).

By synthesizing our results (and those of our previous studies^36,37,43^) with conclusions from prior geochemical taphonomy studies, we propose the following regarding the two most commonly encountered REE-depth profiles in fossil bones predicted to have the highest potential for molecular recovery: "simple" and "moderate" diffusion (Fig. 5). "Simple diffusion" profiles (and the diagenetic histories that produce them) are most likely to occur in bones that: (1) have a thicker and denser cortex (this study, and refs.^24,28,29^); (2) were buried rapidly (this study); (3) were buried in low-permeability sediments (e.g., claystone, sandstone cemented in early diagenesis; this study, and refs.^61,65,67^), and/or; (4) were buried in dry/semi-dry depositional environments^68^. "Moderate diffusion" profiles (and the diagenetic histories that produce them) are most likely to occur in bones that: (1) have a thinner cortex or high porosity^12,26,30,65,69^; (2) were exposed for an extended time (> 10 year, based on weathering) before burial^8^; (3) were preserved in high-permeability sediments (e.g., coarse-grained sandstone, unconsolidated sediments^65^), or; (4) were buried in wet/moist environments^60,64,66,68^.

Though it is tempting to suggest that bones with "simple diffusion" profiles should be the best candidates for biomolecular analyses, the current dataset in support of this claim is only one bone (or nine bones from one facies [mudstone] at one locality if soft tissue recovery is instead the criterion; cf. ref.^36^). Moreover, two fossil bones exhibiting double medium diffusion REE profiles^43^ have also been found to yield cellular and soft tissue microstructures^36^, indicating that specimens with other profile types may also be productive targets. Therefore, fossil bones buried in a wide range of other facies (e.g., limestone, sandstone, unconsolidated sediments) and exhibiting many other types of REE profiles (e.g., irregular, leached, over-steepened in Fig. 5) must be tested for soft tissue and biomolecular preservation to fully evaluate the reliability of our predictions.

It is important to note that although we performed the analyses herein on a well-preserved dinosaur bone from the Hell Creek Formation (a suite of strata already known to yield exquisite fossils at the molecular level^14–17,32,56^), reports of successful recovery of osteocytes, blood vessels, and other soft tissues within bone are becoming more frequent (e.g.,^17,18,45,56^), even from recently-oxidized and sun-bleached float bone fragments^36^. Therefore, even a specimen whose preservation quality visually appears “mediocre” at first glance may be worth examination via REE analyses in order to judge its molecular potential. Trace element analyses are an inexpensive, minimally-destructive way to quickly assess the extent of chemical alteration that any fossil has endured.

Despite the advances in understanding we have developed in this study, the relationship between REE profiles and biomolecular recovery potential remains far from clear. Bone tissue type, structure, and location of biomolecules within cells and tissues are also key variables that must be considered, as recovery of cells and/or soft tissues by demineralization appears positively correlated with biomolecular recovery (this study and refs.^1,4,15,17,36,45^). Extensive alteration by processes such as leaching or secondary uptake phases likely decrease soft tissue and biomolecular preservation potential, but the specimens we have analyzed at SRHS do not shed light on these expectations. Protracted, late-diagenetic uptake of REE will provide erroneous isotopic age estimates for fossil bones, but it does not affect the potential utility of REE profiles as a biomolecular preservation proxy. Thus, in conclusion, all available evidence suggests that REE profiles may be useful proxies for biomolecular recovery when considered alongside a framework of traditional taphonomic and sedimentologic context.

## Materials and methods

### Fossil samples

Sixteen SRHS *Edmontosaurus annectens* bones were previously shown to yield microstructures morphologically consistent with vertebrate osteocytes, blood vessels, and fibrous matrix upon demineralization with 0.5 M ethylenediaminetetraacetic acid (EDTA) pH 8.0^36^. We have reported the trace element composition of nine of those specimens elsewhere^43^ and further examine one of them (fibula SRHS-DU-231) here for biomolecular preservation. All nine bones are morphologically well-preserved: they are complete and exhibit no weathering or abrasion^37^.

### Methods

Methods used for trace element analyses and biomolecular assays are detailed in Ullmann et al.^43^ and the Supporting Information of this paper, respectively. Briefly, cortical bone samples were collected immediately following discovery of bones in the field, and were stored in sterile glass jars over Silicagel desiccant beads until analysis. The trace element composition of each specimen was determined previously by laser ablation-inductively coupled plasma mass spectrometry (LA-ICPMS) on transverse thick sections^43^. Herein, we employed three independent assays to test for the presence of collagen I epitopes in demineralized tissues and chemical extracts from SRHS-DU-231: polyacrylamide gel electrophoresis (PAGE) with silver-staining, enzyme-linked immunosorbant assays (ELISA), and in situ immunofluorescence. ELISA is the most sensitive of these techniques, in which antibodies bind to epitopes in native confirmation in extracts^44^. PAGE separates components of extracts by molecular weight, and though less sensitive than ELISA, this technique enables recognition of denatured epitopes^44^. Immunofluorescence is the least sensitive of these techniques but provides the advantage of localizing epitopes of specific proteins in native confirmation to tissues^44^. All protein extractions, PAGE, and immunoassays were conducted in a laboratory dedicated to biomolecular analyses of fossils at North Carolina State University (NCSU) in which tissues of modern organisms are prohibited.

## Supplementary information

Supplementary information.

## Data Availability

All data generated and analyzed during this study are included in this published article and its Supporting Information file.
