# Supplementary material for: Molecular tests support the viability of rare earth elements as proxies for fossil biomolecule preservation
Source: Sci Rep. 2020 Sep 23;10:15566. doi: 10.1038/s41598-020-72648-6 (PMC7511940; doi:10.1038/s41598-020-72648-6)
Supplement: Supplementary file 1 — Supplementary information. [file 41598_2020_72648_MOESM1_ESM.docx]

Supporting Information

Scientific Reports

**Molecular tests support the viability of rare earth elements as proxies for fossil biomolecule preservation**

Paul V. Ullmann^1^*, Kristyn K. Voegele^1^, David E. Grandstaff^2^, Richard D. Ash^3^, Wenxia Zheng^4^, Elena R. Schroeter^4^, Mary H. Schweitzer^4,5,6,7^, Kenneth J. Lacovara^1^

^1^Department of Geology, Rowan University, Glassboro, New Jersey, United States of America

^2^Department of Earth and Environmental Science, Temple University, Philadelphia, Pennsylvania, United States of America

^3^Department of Geology, University of Maryland, College Park, Maryland, United States of America

^4^Department of Biological Sciences, North Carolina State University, Raleigh, North Carolina, United States of America

^5^Department of Geology, Lund University, Lund, Sweden

^6^North Carolina Museum of Natural Sciences, Raleigh, North Carolina, United States of America

^7^Museum of the Rockies, Montana State University, Bozeman, Montana, United States of America

*Corresponding author

This supplement provides: 1) details on our methodology; 2) PAGE with silverstain results for ABC extracts (S1 Fig.); 3) an additional replicate of fossil ELISA results (S2 Fig.), presenting similar results to those in Fig. 3 of the main text, and; 4) a comparison of fossil versus modern *Alligator* ELISA results at the same time point (S3 Fig.).

**Methods**

**Sample preparation**

All samples were collected during the summer of 2012 while wearing nitrile gloves to limit contamination for ensuing soft tissue and biomolecular analyses (see Ullmann et al., 2017, fig. 5 for a quarry map of specimen locations). Samples of cortical bone were collected immediately following discovery and exposure of bones in the field. A dedicated, autoclaved chisel was gently hammered into bone shafts to extract small fragments of cortical bone. These samples were wrapped in autoclaved aluminum foil and stored in autoclaved glass canning jars over Silicagel desiccant beads until analysis in the laboratory.

**Biomolecular analyses**

Analyses of fossil samples were performed in a dedicated molecular paleontology laboratory at North Carolina State University (NCSU) in which tissues of modern organisms are prohibited (see Schroeter et al., 2017, for additional details on lab conditions). All fossil biomolecular analyses were performed with dedicated, isolated instruments, reagents, buffers, and sterilized laboratory supplies while wearing personal protective equipment to prevent contamination.

As a modern control, cortical bone from the limb bones of an extant archosaur, the American alligator (*Alligator mississippiensis*), were analyzed in a separate, isolated lab with separate, dedicated instruments. Modern *Alligator* bones were defleshed with a sterile scalpel then degreased in a solution of 10% Shout^®^ for 2–3 days on a rocker before being stored at -20°C until analysis (Voegele, 2016). Fossil samples never entered this room, and modern control samples and neither associated laboratory supplies or personal protective equipment ever entered the dedicated "ancient" laboratory. Three replicates were completed of each assay unless otherwise noted.

**Protein extractions**

Over the course of three years, two different protein extractions were performed. Initially, we followed a sequential, demineralization-based immunoprecipitation extraction developed by Schroeter (2013; modified from Zheng and Schweitzer, 2012). In brief, aliquots of two grams of cortical bone were ground to fine powder (< 1 mm grain size) in sterilized mortar and pestles and added to separate 10 ml spin columns (Pierce) in 50 ml centrifuge tubes. Aliquots of sediment were also separately ground to serve as negative controls, and one additional spin column per column of bone powder was left empty to serve as a buffer (“blank”) control. Columns were demineralized overnight at room temperature on a rocker with 10 ml of 0.6 M hydrochloric acid (HCl), then centrifuged the following day to collect an ‘HCl extract’. Demineralization was then continued with 10 ml of 4 M guanidine hydrochloride (GuHCl) in 0.05 M Tris pH 7.4, again incubating (this time at 65°C) overnight on a rocker. Columns were then centrifuged the following day to collect a ‘GuHCl extract’. Protein precipitation began with centrifugation to pelletize any remaining undissolved solids. Resulting supernatants were then decanted into new 50 ml tubes for immunoprecipitation, which was performed for 1–1.5 hr at 4°C with 2.5 ml of 100% trichloracetic acid (TCA) for each HCl extract or overnight at -20°C with 25 ml of 100% ethanol for each GuHCl extract. Precipitated HCl extracts were washed the following day by three rounds of centrifugation, decanting, and rinsing of the pellet with 5 ml of 100% acetone. Precipitated GuHCl extracts were washed by three rounds of centrifugation, decanting, and rinsing of the pellet with 5 ml of 90% ethanol. After final centrifugation and decanting, tubes were inverted over paper towels in a laminar flow hood to dry overnight (at room temperature). Finalized extract tubes were then sealed and stored at -20°C or -80°C until analysis. ‘GuHCl extract’ fractions are expected to contain the majority of the extracted collagen.

For later analyses, we performed a sequential extraction protocol recently developed by one of us (ERS) and successfully used by Voegele (2016) to recover collagen I from a fossil bone. As in the GuHCl extraction above, bone and sediment were ground into fine powders with separate, sterilized mortars and pestles, but now 1g of each powder was added directly to separate 50 ml centrifuge tubes (without spin columns); one tube per tube of bone powder was again left empty to serve as a buffer/"blank" control. 40 volumes of 0.6M HCl were then added and tubes were incubated overnight on a rocker at 4°C. After centrifuging, each supernatant was collected in a new 50 mL centrifuge tube and the remaining pellet was washed twice with 10 mL Epure water, centrifuged, and the supernatant added to the new tube from above to collect an 'HCl extract'. 40 volumes of 0.05M ammonium bicarbonate (ABC) was then added to the remaining pellet, which was vortexed and incubated overnight at 65°C with rocking. Extracts were finalized the next day by centrifuging and collection of each supernatant in a new, separate tube as an 'ABC extract'. HCl extracts were precipitated with TCA and acetone washes as in the Schroeter (2013) protocol outlined above. ABC extracts, which are expected to contain the majority of the collagen extracted, were dried in a speed vacuum and stored at either -20°C or -80°C depending on the amount of time until analysis. Because post-extraction yields from these protocols include salts from buffers in addition to extracted protein (Cleland et al. 2012), concentrations are reported relative to amounts of pre-extracted bone mass (as in Voegele, 2016).

**Polyacrylamide gel electrophoresis (PAGE)**

We followed the protocols of Zheng and Schweitzer (2012) for PAGE and silver-staining, with the addition of treatment of ABC extracts with an iron chelator solution, pyridoxal isonicotinoyl hydrazone (PIH) prior to electrophoresis due to their imparting pigment to gels in our initial tests. For this treatment, ABC extracts were divided into aliquots each comprising 200 mg of pre-extracted bone in 1.5 ml microcentrifuge tubes and incubated overnight (with rocking at room temperature) in 100 µl of 10 mM PIH/50 mM NaOH (a modification of the protocol developed by Schweitzer et al., 2013). The extracts were then lyophilized and solubilized as below for PAGE.

GuHCl and (PIH-treated) ABC extracts were solubilized in 1X Phosphate Buffered Saline (PBS) and combined with an equal volume of 2X Laemmli buffer with 0.1 M dithiothreitol (DTT), to make a final loading volume of 40 μL per lane. Samples were then denatured at 95°C and centrifuged to pelletize any debris. GuHCl extracts were run at 4 mg of yield and ABC extracts were run as the yield from 100–200 mg of pre-extracted mass per lane in 15% polyacrylamide running gels (8% for GuHCl extracts)/5% stacking gels. These loadings are significantly greater than those employed for modern *Alligator* extracts collected in a separate lab (see below) due to expected degradation and diminishment of organic material in bones of Mesozoic age (cf., Schweitzer et al., 2007a, 2009). For ABC extracts, the remaining bone pellet was resuspended with 1X PBS + 2X Laemmli/0.1M DTT solution and loaded in an additional lane; GuHCl extracts did not leave a residual pellet to resuspend. Electrophoresis was then run for 1 hr at 50 mA. Gels were then fixed in 50% methanol/5% acetic acid at room temperature and washed with 50% methanol then Epure water. Gels containing ABC extracts were digitally scanned between two transparency sheets on a Cannon MX300 scanner to capture a 'pre-development' image of any pigmentation imparted from iron, humics, or other substances in the extracts; this was not necessary with gels containing GuHCl extracts at no pre-development coloration of these gel occurred. Gels were then sensitized with 0.2% sodium thiosulfate and incubated in 0.1% silver nitrate at room temperature for 30 min. Following two brief rinses with Epure water, gels were developed with multiple changes of 0.04% formalin in 2% sodium carbonate until staining became apparent (~ 10 min.). Development was terminated with 5% acetic acid, then gels were rinsed with Epure water and digitally scanned as above. For gels containing ABC extracts, a positive result was deemed present only when markedly obvious darkening occurred compared to a 'pre-development' image of the same gel. Control lanes included the surrounding sediment, extraction blanks, and extract resuspension buffers (1X PBS + 2x Laemmli/0.1M DTT).

Modern *Alligator* extracts, extracted with the same protocols in a separate NCSU facility, were added to 15% or 8% polyacrylamide running gels/5% stacking gels at 20 µg/lane. As these samples did not color the gel prior to development, no 'pre-development' images were acquired.

**Enzyme-linked immunosorbant assay (ELISA)**

ABC extracts were resuspended in 1X PBS to a concentration of 1 g of pre-extracted bone per 500 µl, centrifuged to pelletize any remaining insoluble particulates, and plated (at 100 µl/well) on a 96-well Immulon 2HB U-bottom microtiter plate (Thermo Scientific). Solutions were discarded after 4 hr incubation at room temperature, and after each of the following steps (to remove any unbound antigens or antibodies). The plate was then incubated overnight at 4°C in 200 µl/well of ELISA blocking buffer (5% bovine serum albumin in 1X PBS with 0.005% Tween 20 and 2% Thimersol) to avert non-specific binding. Polyclonal rabbit anti-chicken collagen I antibodies (U.S. Biological C7510-13B), diluted to a concentration of 1:400 in blocking buffer, were then added to a subset of wells, whereas blocking buffer was added to control wells. After 4 hr incubation at room temperature, the plate was washed in ELISA wash buffer (10% PBS diluted in Epure water with 0.1% Tween 20) and dried. Wells were then incubated for 2 hr in secondary antibodies (alkaline phosphatase-conjugated goat anti-rabbit IgG [H+L], Invitrogen G-21079) diluted 1:2000 in 1X PBS. The plate was then washed, dried, and 100 µl/well reading substrate (made by adding one tablet of 0.5 mM MgCl2 + p-nitrophenylphosphate [Sigma N-9389] to 10 ml of 9.8% diethanolamine) was added. Absorbance values were read using a Molecular Devices Spectra Max Plus microplate reader (for ancient samples) or a Molecular Devices THERMOmax (for extant *Alligator* samples), both using the proprietary software Softmax Pro 4.8, at the following time intervals: 0, 10, 20, 30, 40, 50, 60, 90, 120, 150, 180, 210, and 240 min. This protocol was also used for modern *Alligator* extracts, with the only difference being plating of modern antigens at 0.6 μg/well. Other ancient controls included the surrounding sediment, resuspension buffer and extraction blanks (to test for contaminants in laboratory reagents), and secondary-only wells (which never received the primary antibody, to test for non-specific binding of secondary antibodies).

**Tissue embedding procedure**

We followed the fossil tissue embedding and immunofluorescence procedures of Schweitzer et al. (2007a, 2009); a step-by-step guide to these protocols is provided by Zheng and Schweitzer (2012). In brief, a roughly cubic centimeter-size cortical fragment of fibula SRHS-DU-231 was decalcified with freshly prepared 0.5 M disodium ethylenediaminetetraacetic acid (EDTA) pH 8.0 in a sterile six well plate. EDTA was exchanged daily for two weeks, then freed demineralization products were collected in sterile 1.5 ml centrifuge tubes, washed with Epure water, transferred to 15 ml tubes, and fixed with 4 ml of 10% neutral buffer formalin pH 7.2 for one hour. After brief washes in Epure water and 1X PBS, samples were transferred to a new 15 ml tube and dehydrated in 70% ethanol. Resin impregnation involved incubation (for one hour) in a 2:1 solution of 70% ethanol and LR White^TM^ (Electron Microscopy Services) followed by two incubations in pure LR White^TM^. A micropipette and a sharpened wooden dowel were then used to transfer demineralization products into 0.95 ml gelatin capsules and final embedding was accomplished by filling capsules with pure LR White^TM^ and polymerizing at 60°C for 48 hours.

Demineralization and embedding of modern *Alligator* tissues were performed following the same protocols (in a separate laboratory), with one exception: demineralization yielded an intact mass of extracellular matrix. Therefore, a small fragment of this mass was extracted and minced with a scalpel on a sterilized lab plate. Sterilized tweezers were then used to transfer these minced tissue fragments into a 1.5 ml centrifuge tube for initial washing.

**Sectioning and immunofluorescence**

220–230 nm sections were cut (using separate diamond knives for modern and ancient tissues) with a Leica EM UC6 ultramicrotome and added to six-well Teflon printed slides (Electron Microscopy Services). Slides were initially dried for 3–4.5 hours on a slide warmer, then continued drying overnight at 45°C to adhere tissue sections to the slide.

Antigen retrieval, blocking, and antibody incubations were then performed as follows, with the volume of each solution added being 100 μl/well. Antigen retrieval was initiated by incubation with 25 μg/ml proteinase K (Roche) in 1X PBS at 37°C for 15 minutes. Following two washes with PBS pH 7.4, antigen retrieval was continued by three incubations in 0.5 M EDTA pH 8.0. After two PBS washes as above, autofluorescence was quenched by two incubations in 1 mg/ml NaBH_4_. After another two washes, slides were placed in a humidity chamber for all remaining incubations. First, sections were incubated for 2 hr in 4% normal goat serum in PBS pH 7.4 to inhibit non-specific antibody binding. Select wells were then incubated (overnight at 4°C) with polyclonal rabbit anti-chicken collagen I antibodies (Millipore AB752P) diluted 1:40 in a primary dilution buffer (PDB = 0.1% bovine serum albumin/0.1% cold fish skin gelatin/0.5% Triton X-100/0.05% sodium azide/0.01 M PBS pH 7.3), chicken collagen-inhibited antibodies (same as above, at ~6 mg of lyophilized chicken collagen/ml in PDB, prepared in the "modern" laboratory), or in solely PBS pH 7.4 (for wells to serve as secondary-only controls). Successful binding of antibodies raised against chicken collagen I to *Alligator* collagen I has been found previously (Schroeter, 2013), confirming the highly conserved nature of archosaurian collagen I. Further, successful binding with an archosaur (*Alligator*) more distantly related to chicken than *Edmontosaurus* signifies that these antibodies should be an appropriate choice for detection of collagen in nonavian dinosaurs.

Following two washes each with PBS pH 7.4/0.5% Tween 20 (PBS-T) then PBS pH 7.4, all wells were incubated for 2 hr at room temperature with biotinylated goat anti-rabbit IgG H+L antibodies (Vector BA-1000) diluted 1:333 in a secondary dilution buffer (SDB = 0.01 M PBS pH 7.2/0.05% Tween 20). After four washes as above, wells were incubated for 1 hr in the dark in fluorescein avidin D (FITC; Vector) diluted 1:1000 in SDB. After four final washes, slides were mounted with 5 μl/well of VectaShield H-1000 mounting medium. Cover slips were applied and slides were stored in the dark until imaging later the same day or the following day at 40X using a Zeiss Axioskop 2 Plus microscope with a Zeiss Axiocam MRC5 camera. Various exposure times were tested spanning 50–200 ms, but only 200 ms images are presented here.

Digestion assays were performed for select wells by incubation in 1 mg/ml collagenase A (Roche) in Dulbecco’s PBS prior to antigen retrieval with proteinase K. Digestions were performed at 37°C in a humidity chamber for either 1 hr (with changes each 20 min.), 3 hr (with changes each hour, then each 20 min. for the final hour), or 6 hr (with changes each hour, then each 20 min. for the final hour). Digestion was not continued longer than 6 hr to avoid potential non-specific degradation of collagen by any trace contaminant proteases in the collagenase A solution as prepared by Roche (e.g., clostripan, a trypsin-like activity, and a neutral protease; Roche Applied Science, 2012). After digestion, slides were washed twice in PBS pH 7.4. Antigen retrieval then proceeded with proteinase K as described above.


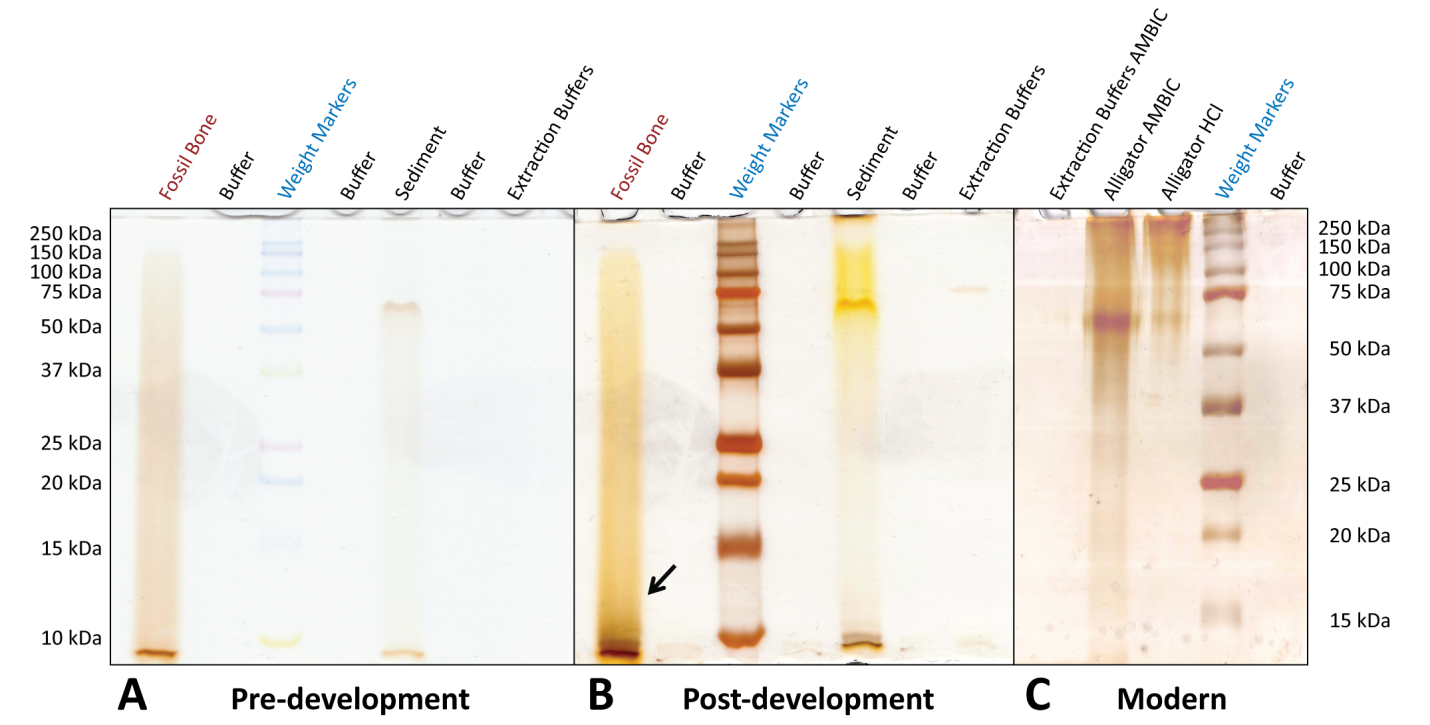


**S1 Fig. Polyacrylamide gel electrophoresis with silver staining of fossil and modern ABC extracts.** (A) "Pre-staining" image of a gel run with PIH-treated fossil extracts prior to incubation with silver nitrate. Fossil bone (*Edmontosaurus* fibula SRHS-DU-231) and sediment ABC extracts were loaded as the yield from 200 mg of pre-extracted mass/lane and extract resuspension buffers were run as a negative control. (B) "Post-staining" image of the same gel taken after development with silver nitrate. Whereas dark brown-orange staining is seen in the form of a smear at low molecular weights in the fossil bone lane (arrow), a yellow-orange smear is seen at high molecular weights in the sediment lane. The origin of the faint band at approximately 65–70 kDa in the extraction buffers lane is uncertain; however, it occurs at the same molecular weight as the band in the sediment lane, suggesting it may owe to slight spillover of the sediment extracts during loading. (C) Silver stain of modern control *Alligator* extracts loaded at 20 µg/lane.





**S2 Fig. Additional replicate of enzyme-linked immunosorbant assay (ELISA) results.** Fossil bone (*Edmontosaurus* fibula SRHS-DU-231) and sediment ABC extracts were plated at 200 mg of pre-extracted mass/well, and extract resuspension buffers were plated as a negative control. White columns represent absorbance values at 240 min with incubation in rabbit anti-chicken collagen I antibodies at a concentration of 1:400. Dark gray columns represent absorbance values for accompanying secondary-only controls. Error bars represent one standard deviation from the mean absorbance value for each sample. Though absorbance values are less than the replicate presented in Fig. 3 of the main text, readings meet the general ELISA criterion of twice background for a positive result (e.g., Ostlund et al., 2001; Appiah et al., 2012).


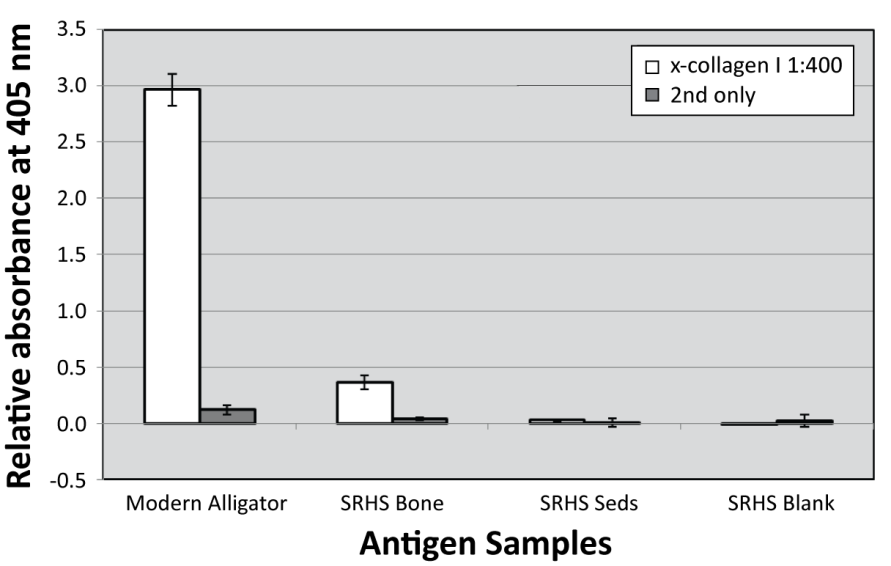


**S3 Fig. Comparison of enzyme-linked immunosorbant assay results for ancient extracts and the modern control.** Modern *Alligator* ABC extracts were run on a separate plate in a separate facility at a concentration of 0.6 µg/well. As presented in Fig. 3 of the main text, fossil bone (*Edmontosaurus* fibula SRHS-DU-231) and sediment ABC extracts were plated at 200 mg of pre-extracted mass/well, and extract resuspension buffers were plated as a negative control. White columns represent absorbance values at 120 min with incubation in rabbit anti-chicken collagen I antibodies at a concentration of 1:400. Dark gray columns represent absorbance values for accompanying secondary-only controls. Error bars represent one standard deviation from the mean absorbance value for each sample.

**REFERENCES**

Appiah AS, Amoatey HM, Klu GYP, Afful NT, Azu E, Owusu GK. Spread of African cassava mosaic virus from cassava (*Manihot esculenta* Crantz) to physic nut (*Jatropha curcas* L.) in Ghana. Journal of Phytology. 2012; 4: 31–37.

Cleland TP, Voegele KK, Schweitzer MH. Empirical evaluation of methodology related to the extraction of bone proteins. PLoS ONE. 2012; 7: e31443.

Ostlund EN, Crom RL, Pedersen DD, Johnson DJ, Williams WO, Schmitt BJ. Equine west nile encephalitis, United States. Emerging Infectious Diseases. 2001; 7: 665–669.

Roche Applied Science. Instructions for use. 2012; REF 10103578001. Available:

<https://pim-eservices.roche.com/LifeScience/Document/4bd132eb-06ee-e311-98a1-00215a9b0ba8>

Schroeter ER. The morphology, histology, and molecular preservation of an exceptionally complete titanosaur from southernmost Patagonia. Ph.D. Dissertation, Drexel University. 2013. Available: <https://idea.library.drexel.edu/islandora/object/idea%3A6996>

Schroeter ER, DeHart CJ, Cleland TP, Zheng W, Thomas PM, Kelleher NL, Bern M, Schweitzer MH. Expansion for the *Brachylophosaurus canadensis* collage I sequence and additional evidence of the preservation of Cretaceous protein. Journal of Proteome Research. 2017; 16: 920–932.

Schweitzer MH, Suo Z, Avci R, Asara JM, Allen MA, Arce FT, et al. Analyses of soft tissue from *Tyrannosaurus rex* suggest the presence of protein. Science. 2007a; 316: 277–280.

Schweitzer MH, Zheng W, Organ CL, Avci R, Suo Z, Freimark LM, et al. Biomolecular characterization and protein sequences of the Campanian hadrosaur *B. canadensis*. Science. 2009; 324: 626–631.

Schweitzer MH, Zheng W, Cleland TP, Bern M. Molecular analyses of dinosaur osteocytes support the presence of endogenous molecules. Bone. 2013; 52: 414–423.

Ullmann PV, Shaw A, Nellermoe R, Lacovara KJ. Taphonomy of the Standing Rock Hadrosaur Site, Corson County, South Dakota. PALAIOS. 2017; 32: 779–796.

Voegele KK. Osteological, mylogical, and biomechanical investigations of the sauropod dinosaur *Dreadnoughtus schrani* and molecular paleontological investigation of the marine crocodile *Thoracosaurus neocesariensis*. Ph.D. Dissertation, Drexel University. 2016. Available: <https://idea.library.drexel.edu/islandora/object/idea%3A7628>

Zheng W, Schweitzer MH. Chemical analyses of fossil bone. In: Bell LS, editor. Forensic microscopy for skeletal tissues: methods and protocols. New York: Humana Press; 2012. pp. 153–172.
